# High‐fat diet affects skeletal muscle mitochondria comparable to pressure overload‐induced heart failure

**DOI:** 10.1111/jcmm.15325

**Published:** 2020-05-04

**Authors:** Estelle Heyne, Andrea Schrepper, Torsten Doenst, Christina Schenkl, Katrin Kreuzer, Michael Schwarzer

**Affiliations:** ^1^ Department of Cardiothoracic Surgery Jena University Hospital – Friedrich Schiller University of Jena Jena Germany

**Keywords:** heart failure, high‐fat diet, interfibrillar/subsarcolemmal mitochondria, pressure overload, skeletal muscle

## Abstract

In heart failure, high‐fat diet (HFD) may exert beneficial effects on cardiac mitochondria and contractility. Skeletal muscle mitochondrial dysfunction in heart failure is associated with myopathy. However, it is not clear if HFD affects skeletal muscle mitochondria in heart failure as well. To induce heart failure, we used pressure overload (PO) in rats fed normal chow or HFD. Interfibrillar mitochondria (IFM) and subsarcolemmal mitochondria (SSM) from gastrocnemius were isolated and functionally characterized. With PO heart failure, maximal respiratory capacity was impaired in IFM but increased in SSM of gastrocnemius. Unexpectedly, HFD affected mitochondria comparably to PO. In combination, PO and HFD showed additive effects on mitochondrial subpopulations which were reflected by isolated complex activities. While PO impaired diastolic as well as systolic cardiac function and increased glucose tolerance, HFD did not affect cardiac function but decreased glucose tolerance. We conclude that HFD and PO heart failure have comparable effects leading to more severe impairment of IFM. Glucose tolerance seems not causally related to skeletal muscle mitochondrial dysfunction. The additive effects of HFD and PO may suggest accelerated skeletal muscle mitochondrial dysfunction when heart failure is accompanied with a diet containing high fat.

## 1 INTRODUCTION

The incidence of heart failure is increasing worldwide and strongly affects public health.^1^ Depending on age, the prevalence of heart failure in the general population is around 1%‐4%.^2^ In heart failure patients, disturbed cardiac mitochondrial function contributes to contractile dysfunction.^3^, ^4^, ^5^, ^6^, ^7^ Mitochondrial adenosine triphosphate (ATP) production is responsible for the vast majority of ATP demand not only in the heart, but also in skeletal muscle. It has been shown that skeletal mitochondrial function is compromised in heart failure patients.^8^ Furthermore, it is assumed that skeletal mitochondrial dysfunction is associated with fibre atrophy and therefore may contribute to muscle wasting and finally skeletal myopathy.^9^, ^10^, ^11^ In rats subjected to pressure overload (PO), we have previously shown that mitochondrial skeletal muscle function was impaired when heart failure was pronounced.^12^ Respiratory capacity and activity of complex I as well as complex II were decreased.^12^

Nutritional strategies to treat heart failure have been suggested.^13^, ^14^ High‐fat diet (HFD) has received significant attention for its potentially protective effect under experimental conditions leading to heart failure. For instance, Rennison et al described HFD feeding in rats with coronary artery ligation to increase oxidative phosphorylation and electron transport chain (ETC) complex activities.^15^ Furthermore, HFD feeding led to improvement of left ventricular contractile performance in hypertensive rats^16^ and a survival advantage compared with normal chow (NC) feeding under heart failure conditions has been described.^17^ However, there is no information if HFD feeding under heart failure conditions could maintain or, even though, improve mitochondrial respiration in skeletal muscle.

In this context, it is important to consider that two distinct mitochondrial subpopulations have been identified in muscle. They differ in their subcellular location.^18^ Under many pathological conditions including myocardial infarction, ischemia‐reperfusion or diabetes, the subpopulations are affected differentially.^18^ In the heart, we recently showed that PO‐induced heart failure leads to a greater depression of respiratory capacity in interfibrillar mitochondria (IFM) compared with subsarcolemmal mitochondria (SSM).^19^

Thus, we now assessed mitochondrial function of skeletal muscle when rats were subjected to PO and HFD. We placed special focus on IFM and SSM, aiming to prevent PO‐induced skeletal muscle mitochondrial dysfunction by HFD.

## 2 MATERIALS AND METHODS

### 2.1 Animals

Male Sprague‐Dawley rats were obtained from Charles River (Sulzfeld, Germany) or Janvier (Genest, France), were fed *ad libitum* and kept at 21°C with a light cycle of 12 hours. The use of animals was consistent with the Guide for the Care and Use of Laboratory Animals, published by the National Institutes of Health (NIH Publication no. 85‐23, revised 1996), and the experimental protocols were approved by the local authorities (Thüringer Landesamt für Verbraucherschutz).

After mating, dams were fed NC (V1534 with 9 kJ% fat, 24 kJ% protein and 67 kJ% carbohydrates) or HFD (D12492 with 60 kJ% fat, 20 kJ% protein and 20 kJ% carbohydrates) in a randomized manner until weaning. Detailed diet compositions are listed in Supporting Information S1. There were no major differences in micronutrient content. The respective diet was continuously applied in weanlings. This protocol was chosen to induce reduced insulin and glucose tolerance already in weanlings. At the same time (3 weeks of age), animals of each diet were randomized into two groups and subjected to transverse aortic constriction (TAC) to induce PO or kept as controls. Normal chow animals did not receive a sham operation. Surgery was a very short intervention and as previously published, even after 2 weeks there was no difference between sham operated and control animals without surgery.^20^ The four resulting experimental groups were as follows: NC (n = 7), NC PO (n = 5), HFD (n = 9) and HFD PO (n = 4). All assessments were conducted at 10 weeks after PO (13 weeks of age).

### 2.2 Materials

Chemicals were obtained from Sigma‐Aldrich (Deisenhofen), Merck (Darmstadt), Serva (Heidelberg), Essex (München), Bayer (Leverkusen), Narkodorm‐n (Neumünster), GeriaSan (Heppenheim) and Bio‐Rad (München). Diet was obtained from SSniff (Soest).

### 2.3 Surgical intervention

The model of heart failure^21^ has been described in detail before. Rats of 30‐50 g (3 weeks of age) were anaesthetized with intramuscular ketamine (50 mg/kg) and xylazine (10 mg/kg), intubated with 16‐gauge tubing and ventilated with room air (1 mL, 96/min). A partial median sternotomy and thymectomy were performed. After dissection of the aortic arch, a titanium clip (0.35 mm internal diameter; Pilling‐Weck, Kernen, Germany) was placed around the aorta between the brachiocephalic trunk and the left common carotid artery. The sternotomy was closed with interrupted sutures and the skin closed with running sutures. After vital signs were re‐established, rats were extubated and kept on warming pads for the recovery periods.

### 2.4 Animal care and echocardiography

Rats were weighed and inspected weekly. Echocardiographic examination was performed 10 weeks postoperatively (13 weeks of age) as previously described.^20^ Briefly, animals were anaesthetized with Isoflurane (1.5%). Chests were shavedand the rats were examined in supine position with a 17.5 MHz phased array transducer (RMV716; VisualSonics, Germany). Fractional shortening was determined and two‐dimensional short‐axis views of the left ventricle at the papillary muscle level were obtained. Two‐dimensional‐guided M‐mode tracings were recorded with a sweep speed of 100 mm/s. We determined left ventricular wall thickness (posterior wall thickness) and cavity size in diastole (left ventricular end‐diastolic dimension) by the American Society for Echocardiology leading edge method and averaged values from five measurements for each examination.^20^

### 2.5 Glucose tolerance test

Directly before the glucose^22^ tolerance test was performed as previously described, rats were fasted for 6h. Rats were anaesthetized with Isoflurane (1.5%) for injection procedure as well as blood sampling. For glucose tolerance test, a single dose of 20% glucose (2g/kg) was administered by intraperitoneally injection. One drop of peripheral blood was used at 0, 15, 30, 60 and 120 minutes. Blood glucose (mmol/L) was measured by using a glucometer (Freestyle mini, Abbott; Germany) and blood sugar test stripes (Freestyle; Abbott). Area under the curve was determined for data analyses.

### 2.6 Organ harvesting

At 13 weeks of age, animals were weighed and  sacrificed. Deep anaesthesia was induced using thiopental (150 mg/kg bodyweight)and hearts were explanted and weighed. *Musculus (M.) gastrocnemius* was excised, weighed and prepared for isolation of mitochondria. Both, lungs and liver were excised and weighed. Lung‐to‐bodyweight index and liver‐to‐body index (LiverBI) were calculated as lung wet weight (g), liver wet weight (g), respectively, to bodyweight (kg). Furthermore, both *Musculi solei* and epididymal fat pads were excised and weighed. Length of the left tibia was measured and heart weight as well as muscle weights to tibia length were calculated.

### 2.7 Isolation of mitochondria

Skeletal muscle SSM and IFM were isolated using the procedure of Palmer et al^23^ except that a modified Chappell‐Perry buffer, containing (in mmol/L) 100 KCl, 50 MOPS, 1 EGTA, 5 MgSO47H2O and 1 ATP (pH 7.4 at 4°C), was used for isolation of both mitochondrial populations. The IFM were harvested following treatment with 5 mg/g wet weight trypsin for 10 minutes at 4°C.^19^ Mitochondrial protein concentration was determined by the Bradford method using bovine serum albumin as a standard. Mitochondrial citrate synthase activity was measured in fresh muscle homogenate and isolated mitochondria according to the protocol by Srere.^24^ Mitochondrial yield was calculated as citrate synthase activity recovered in the respective subpopulation in relation to total citrate synthase activity in homogenate.

### 2.8 Mitochondrial respiratory capacity

Oxygen consumption of isolated mitochondria was measured using a Clark‐type oxygen electrode (Strathkelvin) at 25°C. Mitochondria were incubated in a solution containing 80 mmol/L KCl, 50 mmol/L MOPS, 1 mmol/L EGTA, 5 mmol/L KH2PO4 and 1 mg/mL fatty acid‐free bovine serum albumin at pH 7.4. The rate of respiratory capacity was measured using glutamate, pyruvate and malate, palmitoyl‐carnitine and malate, palmitoyl‐CoA, carnitine and malate, succinate and rotenone or tetramethylphenylendiamin as substrates and adenosine diphosphate (ADP) as stimulus. The ADP‐stimulated oxygen consumption (state 3) and the ADP‐limited oxygen consumption (state 4) in the respiratory chamber and the ADP/O ratio (ADP added per oxygen consumed) were determined as previously described.^19^

### 2.9 Determination of isolated complex activities

Mitochondria were treated with 1 mg cholate/mg mitochondrial protein and further prepared according to Rosca et al.^25^ After one cycle of freeze/thaw (−80 to 25°C), ETC complex activities were measured as specific donor‐acceptor oxidoreductase activities.^26^ Complex I was measured as rotenone‐sensitive reduction in 2,6‐dichloroindophenol with NADH as substrate.^27^ Reduction in 2,6‐dichloroindophenol with succinate as substrate assesses complex II.^28^ Complex III activity was determined as antimycin‐A‐sensitive reduction in cytochrome c^28^ using decylubiquinol as substrate, which was prepared as previously described.^29^ Complex IV activity was measured as oxidation of reduced cytochrome c.^30^ Rotenone‐sensitive NADH‐cytochrome c reductase assesses complexes I and III.^25^ Antimycin A‐sensitive succinate‐cytochrome c reductase assesses complexes II and III.^25^

### 2.10 Statistical analysis

Data are presented as means ± SEM. Data were analysed using two‐way ANOVA or Student's *t* test where appropriate. Post‐Hoc comparisons among the groups were performed using the Bonferroni method. Differences among groups were considered statistically significant if *P* < 0.05. Main effects for mitochondrial population, surgery and diet were given in figures and tables. All figures were designed with SigmaPlot.

## 3 RESULTS

**FIGURE 1:**
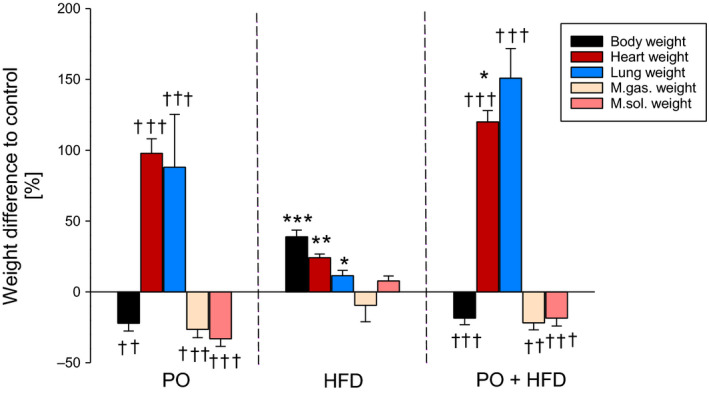
Relative changes in weights of animals 10 wk after pressure overload and with high‐fat diet (HFD) or in combination compared with control. Data are mean + SEM. Normal Chow (NC) n = 7, HFD n = 8‐9, NC + pressure overload (PO) n = 5 and HFD + PO n = 4. *P < 0.05, **P < 0.01, ***P < 0.001 for pressure overload (T), diet (D), interaction (I) or to respective control group of same surgical treatment; ^††^ *P* < 0.01, ^†††^ *P* < 0.001 to group of same diet

In the present and earlier studies, aortic constriction surgery was well tolerated with an operative mortality of 2%.^12^, ^26^ After a 3‐ to 4‐day recovery period, animals demonstrated normal growth. PO and HFD led to differing changes in bodyweight. Figure 1 illustrates the changes induced by PO, HFD or both in total body and individual organ weights (detailed data are shown in Supporting Information S2). PO resulted in a decrease of body and skeletal muscle weight (*M. gastrocnemius* and *M. soleus*), which may be considered as a sign of skeletal muscle atrophy. In contrast, heart and lung weights were increased. HFD resulted in weight gain without affecting skeletal muscle weights. HFD also caused moderate but significant increases in heart and lung weight. HFD in combination with PO attenuated PO‐induced body and skeletal muscle weight loss but aggravated the increase in heart and lung weights.

Table 1 shows echocardiographic parameters (which are consistent with heart and lung weight data), suggesting the presence of heart failure after 10 weeks of PO. PO increased left ventricular posterior wall thickness in diastole (indicating cardiac hypertrophy), increased left ventricular end‐diastolic dimension (indicating cardiac dilation) and increased the ratio of early to late peak velocities flow in late diastole (E/A: indicating diastolic dysfunction). In addition, PO led to decreased fractional shortening as a sign for decreased systolic function. HFD was associated with a trend towards cardiac hypertrophy (12.5%, left ventricular posterior wall thickness in diastole) consistent with the mildly elevated heart weight. There was no further deviation of cardiac function. Adding HFD to PO enhanced hypertrophy, attenuated left ventricular dilatation and also attenuated the impairment of contractile dysfunction.

**TABLE 1:** Echocardiographic parameters of animals after 10 wk of pressure overload and with HFD compared to control

	Control	PO	HFD	PO + HFD	T	D	I
LVPWD [mm]	3.52 ± 0.11	4.92 ± 0.10^†††^	3.96 ± 0.15	6.14 ± 0.27***, ^†††^	***	***	*
LVEDD [mm]	7.83 ± 0.12	8.63 ± 0.12^††^	7.99 ± 0.28	8.13 ± 0.29	*	ns	ns
E/A	1.44 ± 0.05	7.89 ± 0.57^†††^	1.44 ± 0.06	5.47 ± 1.07^†^	***	ns	ns
FS (%)	45.4 ± 0.95	33.4 ± 1.14^†††^	54.1 ± 1.64	44.5 ± 3.51***, ^†^	***	***	ns

Note

Data are mean ± SEM.

Abbreviations: E/A, E‐wave/A‐wave (ratio of peak velocity flow in early diastole to peak velocity flow in late diastole); n = 12‐54; FS, fractional shortening; HFD, high‐fat diet; LVEDD, left ventricular end‐diastolic dimension; LVPWD, left ventricular posterior wall thickness in diastole; PO, pressure overload.

* *P* < 0.05,

*** *P* < 0.001 for pressure overload (T), diet (D), interaction (I) or to respective control group of same surgical treatment;

^†^ *P* < 0.05,

^††^ *P* < 0.01,

^†††^ *P* < 0.001 to group of same diet; ns, non significant.

**FIGURE 2:**
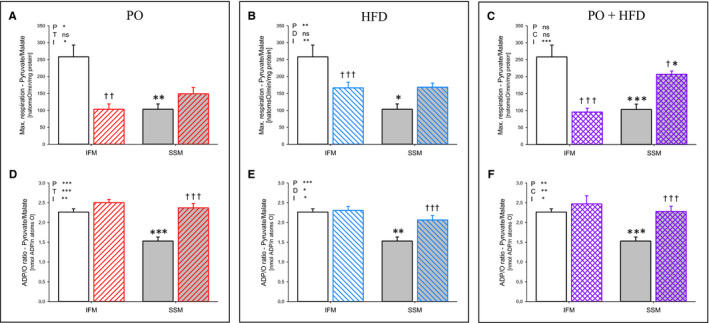
Maximal mitochondrial respiration (state 3, ADP stimulated; A‐C) and ADP/O ratios (D‐F) of interfibrillar mitochondria (white bars) and subsarcolemmal mitochondria (grey bars) with pyruvate and malate of control animals (empty bars) compared to animals subjected to pressure overload (PO) (ascending lines; A, D), compared to animals fed high‐fat diet (HFD) (descending lines; B, E) and compared to animals with PO and HFD (intersecting lines; C, F). Data are mean + SEM. Normal chow (NC) n = 7, HFD n = 8, NC + PO n = 5 and HFD + PO n = 4. *P < 0.05, **P < 0.01, ***P < 0.001 for pressure overload (T), diet (D), interaction (I) or to respective control group of same surgical treatment; ^†^ *P* < 0.05, ^††^ *P* < 0.01, ^†††^ *P* < 0.001 to group of same diet; ns, non significant

Figure 2 shows maximal ADP‐stimulated respiration as well as ADP/O ratio of skeletal muscle mitochondrial subpopulations using pyruvate/malate as substrate. Maximal respiration was about 50% higher in IFM compared with SSM. Pressure overload led to a significant reduction in maximal respiration in IFM. In contrast, maximal respiration showed a trend towards an increase with PO in SSM. The same pattern was found with all other substrates used (Supporting Information S3). ADP/O ratio was higher in IFM compared with SSM in controls, indicating increased coupling of ATP production to O2 consumption in IFM. Pressure overload had no effect on ADP/O ratios in IFM but significantly increased ADP/O ratios in SSM. Unexpectedly, HFD caused identical changes in maximal respiration and ADP/O ratios. Even more so, the effects of PO and HFD on mitochondrial function were additive. In IFM, a 63% decrease in maximal respiration was found and a 100% increase in SSM. The combination did not change the individual impact of PO and HFD on ADP/O ratios.

**FIGURE 3:**
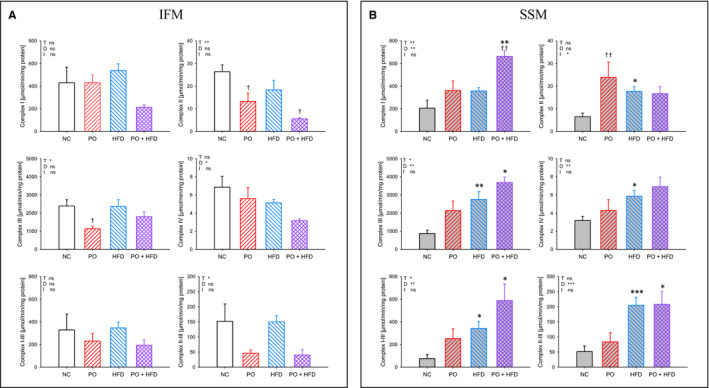
Mitochondrial isolated complex activities of interfibrillar mitochondria (A; white bars) and subsarcolemmal mitochondria (B; grey bars) of control animals (empty bars) compared to animals subjected to pressure overload (PO) (ascending lines), compared to animals fed high‐fat diet (HFD) (descending lines) and compared to animals with PO and HFD (intersecting lines); data are mean + SEM. Normal chow (NC) n = 5‐9, HFD n = 8‐9, NC + PO n = 5‐6 and HFD + PO n = 4. *P < 0.05, **P < 0.01, ***P < 0.001 for pressure overload (T), diet (D), interaction (I) or to respective control group of same surgical treatment; ^†^ *P* < 0.05, ^††^ *P* < 0.01 to group of same diet; ns, non significant

Isolated complex activities are shown in Figure 3. In general, there was a trend towards decreased complex activities in IFM and increased complex activities in SSM. The combination of PO and HFD led to the lowest complex activities in IFM and at the same time to the highest complex activities in SSM and therefore reflected the changes in respiratory capacity.

To account for potential differences in mitochondrial mass, we assessed citrate synthase activity. Citrate synthase activity in *M gastrocnemius* did not change with PO or HFD, indicating no differences in mitochondrial mass (see Supporting Information S4 for details). A combination of PO and HFD resulted again in normal values compared with control. Citrate synthase activity of isolated IFM was reduced with PO as well as with HFD. In contrast, citrate synthase activity of isolated SSM related to protein content was increased with HFD and more pronounced in combination with PO. Mitochondrial yield in IFM was higher with HFD as well as combined with TAC, indicating greater stability of mitochondria.

**FIGURE 4:**
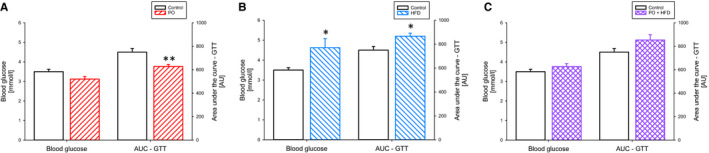
Fasting blood glucose level and glucose tolerance (area under the curve) of control animals (empty bars) compared to animals subjected to pressure overload (PO) (ascending lines; A), compared to animals fed high‐fat diet (HFD) (descending lines; B) and compared to animals with PO and HFD (intersecting lines; c). Data are mean + SEM, normal chow (NC) n = 7, HFD n = 6, NC + PO n = 10 and HFD + PO n = 14. *P < 0.05, **P < 0.01 for pressure overload (T), diet (D), interaction (I) or to respective control group of same surgical treatment

Both, HFD and also PO have been described as modifiers of insulin sensitivity. Figure 4 shows the results of glucose tolerance tests. Fasting blood glucose was unchanged with PO and elevated with HFD. The combination of PO and HFD resulted in normal fasting blood glucose. PO improved glucose tolerance, while HFD impaired it. The effects were additive in that the combination resulted in normal blood glucose and glucose tolerance.

## 4 DISCUSSION

We show here for the first time that HFD causes skeletal muscle mitochondrial dysfunction comparable to that found in PO induced heart failure. The absence of glucose intolerance in PO suggests that glucose intolerance may not be related to skeletal muscle mitochondrial dysfunction in this model. The effects of PO and HFD on mitochondria were additive, which suggests accelerated development of skeletal muscle dysfunction when heart failure is accompanied with a diet containing high fat.

High fat diet feeding has been shown to improve mitochondrial function and/or density.^31^ However, HFD can be both, beneficial as an adaptive response or detrimental, additionally inducing obesity and insulin resistance.^31^ In our investigation, HFD feeding resulted in decreased maximal mitochondrial respiration of IFM in skeletal muscle, whereas maximal mitochondrial respiration of SSM tended to be increased. This was combined with impaired glucose tolerance, indicating a pronounced diabetic phenotype. Our results are consistent with Jørgensen et al, who described significantly reduced succinate‐dependent mitochondrial respiration combined with increased insulin resistance with 1 year of HFD feeding (60% fat).^32^ In contrast, short‐term HFD feeding of 15 days led to increased oxidative capacity.^33^ These observations suggest that the duration of treatment may determine the observed effect of HFD. The changes in our investigation, found with HFD, seem to be rather detrimental than adaptive.

Heart failure patients display functional and structural alterations in skeletal muscle mitochondria.^34^, ^35^ Consistent with our previous results,^36^ we found a significant decrease in maximal respiration in IFM with PO. Additionally, we found a tendency to increased maximal respiration in SSM. Impairment in mitochondrial function and maximal respiration in heart failure seem independent of the model used^37^, ^38^, ^39^ but have only rarely been addressed in IFM and SSM separately. Differentiating between IFM and SSM led to decreased mitochondrial respiration in both mitochondrial populations in dogs with pacing‐induced heart failure.^40^ However, differences in species and the stimulus to induce heart failure do not allow for exact comparison with our study. In our model, PO led to opposite changes in skeletal muscle IFM and SSM, with impairment in IFM function only.

Interestingly, comparison of the influence of HFD and PO on mitochondrial function indicates similar effects of both treatments on skeletal muscle mitochondrial respiratory capacity as well as respiratory efficiency (increased ADP/O). There are no other studies investigating the combined effect of HFD and PO. However, in the context of heart failure, HFD has been suggested to prevent the development and progression of heart failure along with maintenance of mitochondrial content and function.^17^ Unexpectedly, we found additive effects on mitochondrial respiration when PO and HFD were combined. Compared to single treatments, respiratory capacity of IFM was decreased more severely and SSM's respiratory capacity was significantly increased. The reduction in IFM mitochondrial function was not compensated by increased mitochondrial mass since we did not find any changes in citrate synthase activity. Thus, we are the first to show that a HFD in the context of heart failure results in accelerated development of skeletal muscle mitochondrial dysfunction.

The comparable and additive effect of HFD and PO on mitochondrial function raises the question about the underlying mechanism. Our results indicate that one potential mechanism may be excluded: insulin resistance and heart failure are epidemiologically associated,^2^ and insulin resistance is associated with mitochondrial dysfunction.^41^, ^42^ Mostly, insulin resistance is accompanied by glucose intolerance. In this investigation, PO had no effect on glucose tolerance. Furthermore, the combination of PO and HFD resulted in normal glucose response. Thus, glucose intolerance seems not to be a common mechanism for the changes in mitochondrial function found here.

Changes in isolated complex activities may be another potential cause for impaired mitochondrial function.^43^ Indeed, our data indicate that this seems a common and probable mechanism for the comparable and additive effects of PO and HFD. PO or HFD as single treatments led to a decrease in some isolated complex activities in IFM and to an increase in single‐complex activities in SSM. Interestingly, the combination of PO and HFD resulted in the lowest complex activities in IFM and in the highest complex activities in SSM. Changes in complex activities matched the changes in mitochondrial respiration. Thus, we found an association between mitochondrial respiration and complex activities. As a further consequence, the comparable and additive effects of PO and HFD on mitochondrial function seem to be directly related to changes in mitochondrial complex activities.

Another new effect is that HFD and PO show a comparable influence on IFM and SSM but in an opposite direction. First of all, some other studies show no difference in mitochondrial skeletal muscle respiration with HFD^44^ or in skeletal muscle of heart failure patients compared with controls.^45^ In these investigations, mitochondrial subpopulations have not been separately investigated. Instead, the skinned fibre technique has been used where SSM are predominantly assessed. Thus, we speculate that these results may disguise differences in mitochondrial respiration. Further studies should take this into account and separate mitochondrial subpopulations.

Furthermore, it has been suggested that IFM mainly deliver ATP for muscle contraction, whereas ATP derived from SSM is used for maintenance of basic cell function.^19^ In the present study, PO and HFD led to a decrease in mitochondrial respiration in IFM and an increase in SSM. Increased activity of SSM may be seen as a compensatory adaptation to meet ATP demand. The fact that the decrease in mitochondrial function was present in IFM only would support the notion that muscle contractile dysfunction seems to be due to insufficient ATP delivery by IFM. Furthermore, the impairment in IFM function may reduce the total ATP supply for the cell, as IFM represent the majority (80%) of mitochondria within skeletal muscles.^46^ Consequently, mitochondrial impairment with HFD seems to have similar functional relevance and may lead to skeletal myopathy comparable to that found in heart failure patients and experimental models (with mitochondrial dysfunction).^47^, ^48^, ^49^, ^50^, ^51^ We speculate that the observed mitochondrial IFM dysfunction with HFD and PO in combination may contribute to development of accelerated skeletal myopathy. One striking feature is the lack of skeletal muscle fibre type assessment or functional analysis. We speculate that our observed mitochondrial dysfunction may result in skeletal myopathy. Its actual presence, however, is not relevant for our observed findings on mitochondrial function. Furthermore, we did not directly address mechanisms of differential regulation of IFM and SSM. Mitochondrially encoded proteins could be regulated differentially. Differences in protein import,^52^ protein degradation,^53^, ^54^ post‐translational modifications^55^ or mitochondrial dynamics^56^ are potential processes which may lead to different effects of PO and HFD on mitochondrial subpopulations.

The composition of HFD may be an important factor. We used a diet containing 60% fat (mainly lard). Long‐chain saturated fatty acids were present in more than the double amount of long‐chain unsaturated fatty acids. On the one hand, long‐chain saturated instead of unsaturated fatty acids have been described to be involved in lipotoxic pathways.^57^ On the other hand, saturated but not unsaturated fatty acids increased mitochondrial function in rats.^58^ Nevertheless, our results with a high amount of saturated fatty acids suggest a substantial detrimental effect on skeletal muscle. Today, nutrition guidelines for heart failure patients mainly focus on sodium restriction but do not provide specific recommendations for fat intake. The reason may be that only few human nutrition interventional studies exist in this context. Furthermore, the effects of nutrition on skeletal muscle in human heart failure have not been addressed so far. While our results were obtained in rats, the findings may be relevant in humans as well. PO‐induced heart failure in rats is a commonly used model for heart failure induced by hypertension or aortic valve stenosis in patients. It is clinically relevant since heart failure develops with steady progression. Compensated hypertrophy is followed by heart failure with first diastolic and subsequently systolic dysfunction.^59^ Our results suggest that it may be necessary to control heart failure patients diet.

In conclusion, HFD causes skeletal muscle mitochondrial dysfunction comparable to that found in PO heart failure. Furthermore, PO and HFD affect mitochondrial subpopulations adversely. The absence of glucose intolerance in PO may indicate that glucose intolerance is not causally related to skeletal muscle mitochondrial dysfunction in this model. In addition, effects of PO and HFD on mitochondria were additive leading to more severe impairment of IFM. This may suggest accelerated development of skeletal muscle dysfunction when heart failure is accompanied by a diet containing high amounts of fat.

## CONFLICT OF INTEREST

The authors declare that they have no conflict of interest.

## AUTHORS' CONTRIBUTION

EH planned and performed experiments, analysed data and drafted and revised the manuscript. AS, CS and KK collected and analysed data. TD prepared and revised the manuscript. MS designed the study and prepared and revised the manuscript.

## Supporting information

Table S1‐S4

## Data Availability

The data supporting the findings of this study are available from the corresponding author upon reasonable request.

## References

[1] Abraham WT , Fonarow GC , Albert NM , et al. Predictors of in‐hospital mortality in patients hospitalized for heart failure: insights from the Organized Program to Initiate Lifesaving Treatment in Hospitalized Patients with Heart Failure (OPTIMIZE‐HF). J Am Coll Cardiol. 2008;52:347‐356. 18652942 10.1016/j.jacc.2008.04.028

[2] MacDonald MR , Petrie MC , Hawkins NM , et al. Diabetes, left ventricular systolic dysfunction, and chronic heart failure. Eur Heart J. 2008;29:1224‐1240. 18424786 10.1093/eurheartj/ehn156

[3] Rosca MG , Hoppel CL . Mitochondria in heart failure. Cardiovasc Res. 2010;88:40‐50. 20668004 10.1093/cvr/cvq240 PMC3025720

[4] Rosenberg P . Mitochondrial dysfunction and heart disease. Mitochondrion. 2004;4:621‐628. 16120419 10.1016/j.mito.2004.07.016

[5] Garnier A , Zoll J , Fortin D , et al. Control by circulating factors of mitochondrial function and transcription cascade in heart failure: a role for endothelin‐1 and angiotensin II. Circ Heart Fail. 2009;2:342‐350. 19808358 10.1161/CIRCHEARTFAILURE.108.812099

[6] Marin‐Garcia J , Goldenthal MJ . Mitochondrial centrality in heart failure. Heart Fail Rev. 2008;13:137‐150. 18185992 10.1007/s10741-007-9079-1

[7] Hassanpour SH , Dehghani MA , Karami SZ . Study of respiratory chain dysfunction in heart disease. J Cardiovasc Thorac Res. 2018;10:1‐13. 29707171 10.15171/jcvtr.2018.01 PMC5913686

[8] Southern WM , Ryan TE , Kepple K , Murrow JR , Nilsson KR , McCully KK . Reduced skeletal muscle oxidative capacity and impaired training adaptations in heart failure. Physiol Rep. 2015;3:e12353. 25855248 10.14814/phy2.12353 PMC4425959

[9] Harrington D , Anker SD , Chua TP , et al. Skeletal muscle function and its relation to exercise tolerance in chronic heart failure. J Am Coll Cardiol. 1997;30:1758‐1764. 9385904 10.1016/s0735-1097(97)00381-1

[10] Wiener DH , Fink LI , Maris J , Jones RA , Chance B , Wilson JR . Abnormal skeletal muscle bioenergetics during exercise in patients with heart failure: role of reduced muscle blood flow. Circulation. 1986;73:1127‐1136. 3698247 10.1161/01.cir.73.6.1127

[11] Rehn TA , Munkvik M , Lunde PK , Sjaastad I , Sejersted OM . Intrinsic skeletal muscle alterations in chronic heart failure patients: a disease‐specific myopathy or a result of deconditioning? Heart Fail Rev. 2012;17:421‐436. 21996779 10.1007/s10741-011-9289-4

[12] Schrepper A , Schwarzer M , Schope M , Amorim PA , Doenst T . Biphasic response of skeletal muscle mitochondria to chronic cardiac pressure overload—role of respiratory chain complex activity. J Mol Cell Cardiol. 2012;52:125‐135. 22100228 10.1016/j.yjmcc.2011.10.022

[13] Abshire M , Xu J , Baptiste D , et al. Nutritional interventions in heart failure: a systematic review of the literature. J Cardiac Fail. 2015;21:989‐999. 10.1016/j.cardfail.2015.10.004 PMC4666750 26525961

[14] Kerley CP . Nutritional interventions in heart failure: challenges and opportunities. Curr Heart Fail Rep. 2018;15:131‐140. 29623529 10.1007/s11897-018-0388-6

[15] Rennison JH , McElfresh TA , Okere IC , et al. High‐fat diet postinfarction enhances mitochondrial function and does not exacerbate left ventricular dysfunction. Am J Physiol Heart Circ Physiol. 2007;292:H1498‐H1506. 17114240 10.1152/ajpheart.01021.2006

[16] Okere IC , Chess DJ , McElfresh TA , et al. High‐fat diet prevents cardiac hypertrophy and improves contractile function in the hypertensive dahl salt‐sensitive rat. Clin Exp Pharmacol Physiol. 2005;32:825‐831. 16173943 10.1111/j.1440-1681.2005.04272.x

[17] Stanley WC , Dabkowski ER , Ribeiro Jr RF , O'Connell KA . Dietary fat and heart failure: moving from lipotoxicity to lipoprotection. Circ Res. 2012;110:764‐776. 22383711 10.1161/CIRCRESAHA.111.253104 PMC3356700

[18] Hollander JM , Thapa D , Shepherd DL . Physiological and structural differences in spatially distinct subpopulations of cardiac mitochondria: influence of cardiac pathologies. Am J Physiol Heart Circ Physiol. 2014;307:H1‐H14. 24778166 10.1152/ajpheart.00747.2013 PMC4080170

[19] Schwarzer M , Schrepper A , Amorim PA , Osterholt M , Doenst T . Pressure overload differentially affects respiratory capacity in interfibrillar and subsarcolemmal mitochondria. Am J Physiol Heart Circ Physiol. 2013;304:H529‐H537. 23241325 10.1152/ajpheart.00699.2012

[20] Doenst T , Pytel G , Schrepper A , et al. Decreased rates of substrate oxidation ex vivo predict the onset of heart failure and contractile dysfunction in rats with pressure overload. Cardiovasc Res. 2010;86:461‐470. 20035032 10.1093/cvr/cvp414

[21] Schwarzer M , Faerber G , Rueckauer T , et al. The metabolic modulators, Etomoxir and NVP‐LAB121, fail to reverse pressure overload induced heart failure in vivo. Basic Res Cardiol. 2009;104:547‐557. 19294446 10.1007/s00395-009-0015-5

[22] Andrikopoulos S , Blair AR , Deluca N , Fam BC , Proietto J . Evaluating the glucose tolerance test in mice. Am J Physiol Endocrinol Metab. 2008;295:E1323‐E1332. 18812462 10.1152/ajpendo.90617.2008

[23] Palmer JW , Tandler B , Hoppel CL . Biochemical properties of subsarcolemmal and interfibrillar mitochondria isolated from rat cardiac muscle. J Biol Chem. 1977;252:8731‐8739. 925018

[24] Srere PA . [1] Citrate synthase: [EC 4.1.3.7. Citrate oxaloacetate‐lyase (CoA‐acetylating)]. Methods Enzymol. 1969;13:3‐11.

[25] Rosca MG , Vazquez EJ , Kerner J , et al. Cardiac mitochondria in heart failure: decrease in respirasomes and oxidative phosphorylation. Cardiovasc Res. 2008;80:30‐39. 18710878 10.1093/cvr/cvn184 PMC2533423

[26] Schwarzer M , Osterholt M , Lunkenbein A , Schrepper A , Amorim P , Doenst T . Mitochondrial reactive oxygen species production and respiratory complex activity in rats with pressure overload‐induced heart failure. J physiol. 2014;592:3767‐3782. 24951621 10.1113/jphysiol.2014.274704 PMC4192702

[27] Janssen AJ , Trijbels FJ , Sengers RC , et al. Spectrophotometric assay for complex I of the respiratory chain in tissue samples and cultured fibroblasts. Clin Chem. 2007;53:729‐734. 17332151 10.1373/clinchem.2006.078873

[28] Krahenbuhl S , Chang M , Brass EP , Hoppel CL . Decreased activities of ubiquinol:ferricytochrome c oxidoreductase (complex III) and ferrocytochrome c:oxygen oxidoreductase (complex IV) in liver mitochondria from rats with hydroxycobalamin[c‐lactam]‐induced methylmalonic aciduria. J Biol Chem. 1991;266:20998‐21003. 1657942

[29] Kirby DM , Thorburn DR , Turnbull DM , Taylor RW . Biochemical assays of respiratory chain complex activity. Methods Cell Biol. 2007;80:93‐119. 17445690 10.1016/S0091-679X(06)80004-X

[30] Wharton DC , Tzagoloff A . [45] Cytochrome oxidase from beef heart mitochondria. Methods Enzymol. 1967;10:245‐250.

[31] Devarshi PP , McNabney SM , Henagan TM . Skeletal muscle nucleo‐mitochondrial crosstalk in obesity and type 2 diabetes. Int J Mol Sci. 2017;18:831. 10.3390/ijms18040831 PMC5412415 28420087

[32] Jørgensen T , Grunnet N , Quistorff B . One‐year high fat diet affects muscle‐but not brain mitochondria. J Cereb Blood Flow Metab. 2015;35:943‐950. 25757754 10.1038/jcbfm.2015.27 PMC4640253

[33] Iossa S , Mollica MP , Lionetti L , Crescenzo R , Botta M , Liverini G . Skeletal muscle oxidative capacity in rats fed high‐fat diet. Int J Obes Relat Metab Disord. 2002;26:65‐72. 11791148 10.1038/sj.ijo.0801844

[34] Guzmán Mentesana G , Báez AL , Lo Presti MS , et al. Functional and structural alterations of cardiac and skeletal muscle mitochondria in heart failure patients. Arch Med Res. 2014;45:237‐246. 24657595 10.1016/j.arcmed.2014.03.003

[35] Liu SZ , Marcinek DJ . Skeletal muscle bioenergetics in aging and heart failure. Heart Fail Rev. 2017;22:167‐178. 27815651 10.1007/s10741-016-9586-z PMC5352460

[36] Schrepper A , Schwarzer M , Schöpe M , Amorim PA , Doenst T . Biphasic response of skeletal muscle mitochondria to chronic cardiac pressure overload—Role of respiratory chain complex activity. J Mol Cell Cardiol. 2012;52:125‐135. 22100228 10.1016/j.yjmcc.2011.10.022

[37] De Sousa E , Veksler V , Bigard X , Mateo P , Ventura‐Clapier R . Heart failure affects mitochondrial but not myofibrillar intrinsic properties of skeletal muscle. Circulation. 2000;102:1847‐1853. 11023942 10.1161/01.cir.102.15.1847

[38] Rosca MG , Okere IA , Sharma N , Stanley WC , Recchia FA , Hoppel CL . Altered expression of the adenine nucleotide translocase isoforms and decreased ATP synthase activity in skeletal muscle mitochondria in heart failure. J Mol Cell Cardiol. 2009;46:927‐935. 19233197 10.1016/j.yjmcc.2009.02.009

[39] Enache I , Charles AL , Bouitbir J , et al. Skeletal muscle mitochondrial dysfunction precedes right ventricular impairment in experimental pulmonary hypertension. Mol Cell Biochem. 2013;373:161‐170. 23099843 10.1007/s11010-012-1485-6

[40] Marín‐García J , Goldenthal MJ , Moe GW . Abnormal cardiac and skeletal muscle mitochondrial function in pacing‐induced cardiac failure. Cardiovasc Res. 2001;52:103‐110. 11557238 10.1016/s0008-6363(01)00368-6

[41] Boudina S , Sena S , O'Neill BT , Tathireddy P , Young ME , Abel ED . Reduced mitochondrial oxidative capacity and increased mitochondrial uncoupling impair myocardial energetics in obesity. Circulation. 2005;112:2686‐2695. 16246967 10.1161/CIRCULATIONAHA.105.554360

[42] Slattery MJ , Bredella MA , Thakur H , Torriani M , Misra M . Insulin resistance and impaired mitochondrial function in obese adolescent girls. Metab Syndr Relat Disord. 2014;12:56‐61. 24251951 10.1089/met.2013.0100 PMC3942687

[43] Hroudova J , Fisar Z . Control mechanisms in mitochondrial oxidative phosphorylation. Neural Regen Res. 2013;8:363‐375. 25206677 10.3969/j.issn.1673-5374.2013.04.009 PMC4107533

[44] van den Broek NM , Ciapaite J , De Feyter HM , et al. Increased mitochondrial content rescues in vivo muscle oxidative capacity in long‐term high‐fat‐diet‐fed rats. Faseb J. 2010;24:1354‐1364. 20040520 10.1096/fj.09-143842

[45] Mettauer B , Zoll J , Sanchez H , et al. Oxidative capacity of skeletal muscle in heart failure patients versus sedentary or active control subjects. J Am Coll Cardiol. 2001;38:947‐954. 11583863 10.1016/s0735-1097(01)01460-7

[46] Hoppeler H . Exercise‐induced ultrastructural changes in skeletal muscle. Int J Sports Med. 1986;7:187‐204. 3531039 10.1055/s-2008-1025758

[47] Sullivan MJ , Green HJ , Cobb FR . Skeletal muscle biochemistry and histology in ambulatory patients with long‐term heart failure. Circulation. 1990;81:518‐527. 2297859 10.1161/01.cir.81.2.518

[48] Drexler H , Riede U , Munzel T , Konig H , Funke E , Just H . Alterations of skeletal muscle in chronic heart failure. Circulation. 1992;85:1751‐1759. 1315220 10.1161/01.cir.85.5.1751

[49] Mettauer B , Zoll J , Garnier A , Ventura‐Clapier R . Heart failure: a model of cardiac and skeletal muscle energetic failure. Pflugers Arch. 2006;452:653‐666. 16767467 10.1007/s00424-006-0072-7

[50] Haykowsky MJ , Tomczak CR , Scott JM , Paterson DI , Kitzman DW . Determinants of exercise intolerance in patients with heart failure and reduced or preserved ejection fraction. J Appl Physiol. 1985;2015(119):739‐744. 10.1152/japplphysiol.00049.2015 PMC4687865 25911681

[51] Garnier A , Fortin D , Delomenie C , Momken I , Veksler V , Ventura‐Clapier R . Depressed mitochondrial transcription factors and oxidative capacity in rat failing cardiac and skeletal muscles. J Physiol. 2003;551:491‐501. 12824444 10.1113/jphysiol.2003.045104 PMC2343221

[52] Takahashi M , Hood DA . Protein import into subsarcolemmal and intermyofibrillar skeletal muscle mitochondria. Differential import regulation in distinct subcellular regions. J Biol Chem. 1996;271:27285‐27291. 8910303 10.1074/jbc.271.44.27285

[53] Nielsen J , Mogensen M , Vind BF , et al. Increased subsarcolemmal lipids in type 2 diabetes: effect of training on localization of lipids, mitochondria, and glycogen in sedentary human skeletal muscle. Am J Physiol Endocrinol Metab. 2010;298:E706‐E713. 20028967 10.1152/ajpendo.00692.2009

[54] Krieger DA , Tate CA , McMillin‐Wood J , Booth FW . Populations of rat skeletal muscle mitochondria after exercise and immobilization. J Appl Physiol Respir Environ Exerc Physiol. 1980;48:23‐28. 6444398 10.1152/jappl.1980.48.1.23

[55] Sirey TM , Ponting CP . Insights into the post‐transcriptional regulation of the mitochondrial electron transport chain. Biochem Soc Trans. 2016;44:1491‐1498. 27911731 10.1042/BST20160100 PMC5095899

[56] Romanello V , Sandri M . Mitochondrial biogenesis and fragmentation as regulators of muscle protein degradation. Curr Hypertens Rep. 2010;12:433‐439. 20967516 10.1007/s11906-010-0157-8

[57] Listenberger LL , Han X , Lewis SE , et al. Triglyceride accumulation protects against fatty acid‐induced lipotoxicity. Proc Natl Acad Sci U S A. 2003;100:3077‐3082. 12629214 10.1073/pnas.0630588100 PMC152249

[58] Halvorsen B , Rustan AC , Madsen L , et al. Effects of long‐chain monounsaturated and n‐3 fatty acids on fatty acid oxidation and lipid composition in rats. Ann Nutr Metab. 2001;45:30‐37. 11244185 10.1159/000046703

[59] Camacho P , Fan H , Liu Z , He J‐Q . Small mammalian animal models of heart disease. Am J Cardiovasc Dis. 2016;6:70‐80. 27679742 PMC5030387

