# Supplementary material for: High‐fat diet affects skeletal muscle mitochondria comparable to pressure overload‐induced heart failure
Source: J Cell Mol Med. 2020 May 4;24(12):6741–9. doi: 10.1111/jcmm.15325 (PMC7299710; doi:10.1111/jcmm.15325)
Supplement: Supplementary file 1 — Table S1‐S4 [file JCMM-24-6741-s001.doc]

Supporting Information S1: Composition of the diets used in the study (source: Research Diets)

|  | V1534 – Normal Chow | D12492 – High fat diet |
| --- | --- | --- |
| Gross energy [MJ/kg] | 16.2 | 25 |
| Metabolizable energy [MJ/kg] | 13.5 | 21.6 |
| Fat [kJ%] | 9 | 60 |
| Protein [kJ%] | 24 | 20 |
| Carbohydrates [kJ%] | 67 | 20 |
| Crude nutrients [%] |  |  |
| Crude protein (N x 6.25) | 19.0 | 24.4 |
| Crude fat | 3.3 | 34.6 |
| Crude fibre | 4.9 | 6.0 |
| Crude ash | 6.4 | 5.3 |
| Starch | 35.2 | 0.1 |
| Sugar | 5.3 | 9.4 |
| N free extracts | 54.2 | 26.3 |
| Mineral mix [%] | 5.5 | 5.4 |
| Fatty acids [%] |  |  |
| C 12:0 | / | 0.04 |
| C 14:0 | 0.01 | 1.18 |
| C 16:0 | 0.45 | 8.27 |
| C 17:0 | / | 0.38 |
| C 18:0 | 0.09 | 6.06 |
| C 20:0 | 0.01 | 0.04 |
| C 16:1 | 0.01 | 1.33 |
| C 18:1 | 0.62 | 12.29 |
| C 18:2 | 1.76 | 2.53 |
| C 18:3 | 0.23 | 0.34 |
| Amino acid mix [%] | 20.8 | 30 |

Supporting Information S2: Morphometric parameters of animals after 10 weeks of pressure overload and with HFD compared to control

|  | Control | PO | HFD | PO + HFD | T | D | I |
| --- | --- | --- | --- | --- | --- | --- | --- |
| n | 7 | 5 | 9 | 4 | / | / | / |
| Age [weeks] | 14.5 ± 0.1 | 14.7 ± 0.7 | 13.8 ± 0.2 | 13.2 ± 0.2 ** | ns | ** | ns |
| BW [g] | 412 ± 14 | 320 ± 22 †† | 572 ± 20 *** | 355 ± 19 ††† | *** | *** | ** |
| VW [g] | 1.15 ± 0.05 | 2.28 ± 0.12 ††† | 1.43 ± 0. 03 ** | 2.53 ± 0.09 * ††† | *** | *** | ns |
| VW/BW [mg/g] | 2.80 ± 0.07 | 7.16 ± 0.21 ††† | 2.52 ± 0.06 | 7.16 ± 0.3 ††† | *** | ns | ns |
| TL [mm] | 41.4 ± 0.3 | 37.0 ± 1.0 ††† | 43.5 ± 0.3 ** | 38.5 ± 0.3 ††† | *** | ** | ns |
| HW/TL [mg/mm] | 27.8 ± 1.1 | 61.6 ± 2.4 ††† | 32.9 ± 0.8 * | 65.9 ± 2.0 ††† | *** | ** | ns |
| Lung weight [g] | 1.44 ± 0.04 | 2.70 ± 0.54 ††† | 1.60 ± 0.05 * | 3.60 ± 0.3 ††† | *** | ** | ns |
| LBI [mg/g] | 3.49 ± 0.05 | 8.24 ± 1.23 ††† | 2.81 ± 0.08 | 10.2 ± 0.9 * ††† | *** | ns | * |
| LW/HW [g/g] | 1.25 ± 0.03 | 1.16 ± 0.18 | 1.12 ± 0.03 | 1.42 ± 0.11 † | ns | ns | * |
| Liver weight [g] | 1.3 ± 0.63 | 1.24 ± 0.68 | 1.96 ± 1.08 *** | 1.32 ± 0.53 ††† | ** | ** | ** |
| LiverBI [mg/g] | 31.7 ± 0.8 | 39.2 ± 2.9 †† | 34.1 ± 1.1 | 37.2 ± 1.7 | ** | ns | ns |
| M.gas. weight [g] | 5.44 ± 0.25 | 4.0 ± 0.32 ††† | 5.54 ± 0.14 | 4.25 ± 0.27 †† | *** | ns | ns |
| M.gas.BI [g/kg] | 13.2 ± 0.2 | 12.5 ± 0.3 | 9.9 ± 0.4 *** | 11.9 ± 0.2 ††† | ns | *** | ** |
| M.gas./TL [mg/mm] | 131 ± 5 | 108 ± 7 †† | 127 ± 3 | 110 ± 6 † | ** | ns | ns |
| M. sol. weight [mg] | 386 ± 18 | 258 ± 21 ††† | 416 ± 14 | 315 ± 22 ††† | *** | * | ns |
| M.sol.BI [mg/g] | 0.94 ± 0.04 | 0.81 ± 0.02 † | 0.73 ± 0.03 *** | 0.88 ± 0.02 † | ns | ns | *** |
| M.sol./TL [mg/mm] | 9.33 ± 0.41 | 6.96 ± 0.43 ††† | 9.55 ± 0.28 | 8.17 ± 0.51 † | *** | ns | ns |
| E.fat pad [g] | 3.90 ± 0.21 | 1.93 ± 0.31 | 2.10 ± 0.21 *** | 4.65 ± 0.8 ††† | *** | *** | *** |
| E.fat padBI [g/kg] | 9.5 ± 0.5 | 5.9 ± 0.6 | 36.1 ± 2.8 *** | 12.8 ± 1.6 ††† | *** | *** | *** |

Data are mean ± SEM. BW, body weight; VW, ventricular weight; TL, tibia length; LBI, lung to body weight; BI, to body weight, M. Gas, Musculus Gastrocnemius; M. sol., Musculus soleus; E. fat pad, epididymal fat pad. * p<0.05, ** p<0.01, *** p<0.001 for pressure overload (T), diet (D), interaction (I) or to respective control group of same surgical treatment; † p<0.05, †† p<0.01, ††† p<0.001 to group of same diet; n.s. – non significant.

Supporting Information S3: Mitochondrial respiratory function with different substrates of animals after 10 weeks of pressure overload and with HFD compared to NC

|  | NC | PO | HFD | PO + HFD | T | | D | I |
| --- | --- | --- | --- | --- | --- | --- | --- | --- |
| Pyruvate/Malate |  |  |  |  |  | |  |  |
| State 3 - IFM | 258 ± 35 | 133 ± 33 †† | 167 ± 17 * | 96 ± 11 | ** | | * | ns |
| State 3 - SSM | 103 ± 16 | 149 ± 19 | 169 ± 12 ** | 207 ± 9 * | * | | *** | ns |
| State 4 – IFM | 79.5 ± 16.2 | 27.3 ± 6.4 †† | 36.7 ± 5.7 ** | 20.4 ± 2.4 | ** | | * | ns |
| State 4 – SSM | 48.4 ± 4.4 | 37.6 ± 3.4 | 49.7 ± 4.4 | 49.1 ± 3.9 | ns | | ns | ns |
| ADP/O - IFM | 2.26 ± 0.08 | 2.50 ± 0.08 | 2.31 ± 0.10 | 2.47 ± 0.21 | ns | ns | | ns |
| ADP/O - SSM | 1.53 ± 0.10 | 2.37 ± 0.11 ††† | 2.06 ± 0.11 ** | 2.28 ± 0.14 | *** | ns | | * |
| PC/Malate |  |  |  |  |  | |  |  |
| State 3 - IFM | 236 ± 50.5 | 115 ± 26 † | 172 ± 22 | 77 ± 11 | ** | | ns | ns |
| State 3 - SSM | 78 ± 11 | 104 ± 20 | 137 ± 14 * | 142 ± 3 | ns | | ** | ns |
| State 4 – IFM | 63.8 ± 13.7 | 26.3 ± 4.8 † | 36.4 ± 6.0 * | 17.3 ± 1.3 | ** | | ns | ns |
| State 4 – SSM | 48.6 ± 4.2 | 34.4 ± 2.7 | 46.9 ± 4.2 | 45.7 ± 4.2 | ns | | ns | ns |
| ADP/O - IFM | 2.12 ± 0.08 | 2.34 ± 0.08 | 2.09 ± 0.08 | 2.14 ± 0.20 | ns | | ns | ns |
| ADP/O - SSM | 1.12 ± 0.24 | 2.06 ± 0.15 ††† | 1.77 ± 0.09 ** | 1.87 ± 0.12 | ** | | ns | * |
| PCoA/Car/Malate |  |  |  |  |  | |  |  |
| State 3 – IFM | 197 ± 39 | 104 ± 24 † | 132 ± 14 * | 74 ± 8 | * | | ns | ns |
| State 3 – SSM | 63 ± 7 | 87 ± 20 | 119 ± 13 | 148 ± 4 | ns | | ns | ns |
| State 4 – IFM | 59.0 ± 11.4 | 30.3 ± 7.0 † | 35.4 ± 4.7 * | 22.7 ± 3.5 | * | | ns | ns |
| State 4 – SSM | 45.3 ± 3.4 | 35.0 ± 2.6 | 46.7 ± 4.2 | 45.3 ± 5.5 | ns | | ns | ns |
| ADP/O – IFM | 2.24 ± 0.11 | 2.37 ± 0.05 | 2.19 ± 0.11 | 2.27 ± 0.21 | ns | | ns | ns |
| ADP/O – SSM | 1.13 ± 0.24 | 1.93 ± 0.07 †† | 1.83 ± 0.11 ** | 2.12 ± 0.04 | ** | | ** | ns |
| Glutamate |  |  |  |  |  | |  |  |
| State 3 – IFM | 242 ± 46 | 145 ± 21 † | 123 ± 13 ** | 80 ± 10 | * | | ** | ns |
| State 3 – SSM | 105 ± 15 | 165 ± 26 † | 160 ± 10 * | 218 ± 16 † | ** | | ** | ns |
| State 4 – IFM | 52.4 ± 10.8 | 21.5 ± 5.6 † | 29.1 ± 4.7 * | 14.8 ± 1.2 | ** | | ns | ns |
| State 4 – SSM | 49.2 ± 6.4 | 38.5 ± 4.6 | 46.7 ± 4.4 | 44.0 ± 3.6 | ns | | ns | ns |
| ADP/O – IFM | 2.10 ± 0.07 | 2.32 ± 0.07 | 2.05 ± 0.07 | 2.20 ± 0.18 | ns | | ns | ns |
| ADP/O – SSM | 1.41 ± 0.14 | 2.12 ± 0.10 ††† | 1.80 ± 0.08 * | 1.98 ± 0.11 | *** | | ns | * |
| Succinate/Rotenone |  |  |  |  |  | |  |  |
| State 3 - IFM | 363 ± 52 | 222 ± 55 † | 240 ± 25 * | 133 ± 14 | * | | * | ns |
| State 3 - SSM | 105 ± 25 | 211 ± 21 † | 215 ± 22 ** | 270 ± 24 | ** | | ** | ns |
| State 4 – IFM | 100 ± 17 | 61 ± 13 † | 62 ± 7 * | 34 ± 4 | * | | * | ns |
| State 4 – SSM | 49 ± 6 | 73 ± 4 †† | 67 ± 4 * | 77 ± 5 | ** | | * | ns |
| ADP/O - IFM | 1.57 ± 0.08 | 1.35 ± 0.1 | 1.43 ± 0.07 | 1.29 ± 0.17 | ns | | ns | ns |
| ADP/O - SSM | 1.07 ± 0.1 | 1.26 ± 0.09 | 1.3 ± 0.07 | 1.23 ± 0.11 | ns | | ns | ns |
| TMPD/Rotenone |  |  |  |  |  | |  |  |
| State 3 – IFM | 851 ± 120 | 503 ± 128 † | 636 ± 77 | 291 ± 25 † | ** | | ns | ns |
| State 3 – SSM | 227 ± 52 | 449 ± 56 | 540 ± 78 ** | 542 ± 78 | ns | | * | ns |

Data are mean ± SEM. ADP/O – ATP produced per oxygen atom reduced by the respiratory chain, PC – palmitoylcarnitine, PCoA – Palmitoyl-CoA, Car – carnitine, TMPD - tetramethylphenylendiamin. n = 4-9. State 3/State 4 [natoms O/min/mg protein]; ADP/O [nmol ADP/natomsO]; * p<0.05, ** p<0.01, *** p<0.001 for pressure overload (T), diet (D), interaction (I) or to respective control group of same surgical treatment; † p<0.05, †† p<0.01, ††† p<0.001 to group of same diet; ns – non significant.

Supporting Information S4: Skeletal muscle citrate synthase activity and mitochondrial yield of animals after 10 weeks of pressure overload and with HFD compared to control

|  | Control | PO | HFD | PO + HFD | T | D | I |
| --- | --- | --- | --- | --- | --- | --- | --- |
| Citrate synthase [U/mg wet weight] | 33.3 ± 5.6 | 27.5 ± 2.9 | 38.4 ± 3.8 | 32.8 ± 6.2 | ns | ns | ns |
| IFM [U/mg prot.] | 3.21 ± 0.45 | 1.66 ± 0.44 †† | 1.67 ± 0.16 ** | 1.29 ± 0.17 | * | * | ns |
| SSM [U/mg prot.] | 1.16 ± 0.15 | 1.28 ± 0.36 | 1.81 ± 0.15 * | 2.41 ± 0.25 ** | ns | ** | ns |
| Mitochondr. yield |  |  |  |  |  |  |  |
| IFM [%] | 23.7 ± 2 | 21.8 ± 1.8 | 33.6 ± 3.7 * | 35.2 ± 6.1 * | ns | ** | ns |
| SSM [%] | 28.2 ± 4.4 | 21.1 ± 3.1 | 27.4 ± 3.1 | 20.2 ± 2.6 | ns | ns | ns |

Data are mean ± SEM. IFM – interfibrillar mitchondria, SSM – subsarcolemmal mitochondria, n = 4-9.* p<0.05, ** p<0.01, for pressure overload (T), diet (D), interaction (I) or to respective control group of same surgical treatment;†† p<0.01 to group of same diet; ns – non significant.
